# Basal Level p53 Suppresses Antiviral Immunity Against Foot-And-Mouth Disease Virus

**DOI:** 10.3390/v11080727

**Published:** 2019-08-07

**Authors:** Tianliang Zhang, Haotai Chen, Xinsheng Liu, Linlin Qi, Xin Gao, Kailing Wang, Kaishen Yao, Jie Zhang, Yuefeng Sun, Yongguang Zhang, Run Wu

**Affiliations:** 1 College of Veterinary Medicine, Gansu Agricultural University, Lanzhou 730070, China; 2 State Key Laboratory of Veterinary Etiological Biology, OIE/National Foot and Mouth Disease Reference Laboratory, Key Laboratory of Animal Virology of Ministry of Agriculture, Lanzhou Veterinary Research Institute, Chinese Academy of Agricultural Sciences, Lanzhou 730046, China; 3 Jiangsu Co-innovation Center for Prevention and Control of Important Animal Infectious Diseases and Zoonoses, Yangzhou University, Yangzhou 225009, China

**Keywords:** p53, MDM2, foot-and-mouth disease virus, innate immunity, interferon

## Abstract

Tumor suppressor protein p53 (p53) is a master transcription factor that plays key roles in cell cycle arrest, apoptosis, senescence, and metabolism, as well as regulation of innate immunity during virus infection. In order to facilitate their replication and spreading, viruses have evolved to manipulate p53 function through different strategies, with some requiring active p53 while others demand reduction/inhibition of p53 activity. However, there are no clear-cut reports about the roles of p53 during the infection of foot-and-mouth disease virus (FMDV), the causative agent of a highly contagious foot-and-mouth disease (FMD) of cloven-hoofed animals. Here we showed that p53 level was dynamically regulated during FMDV infection, being degraded at the early infection stage but recovered to the basal level at the late stage. Cells depleted of p53 showed inhibited FMDV replication and enhanced expression of the immune-related genes, whereas overexpression of p53 didn’t affect the viral replication. Viral challenge assay with p53 knockout mice obtained similar results, with viral load decreased, histopathological changes alleviated, and lifespan extended in the p53 knockout mice. Together, these data demonstrate that basal level p53 is required for efficient FMDV replication by suppressing the innate immunity.

## 1. Introduction

Tumor suppressor protein p53 (p53) was first reported (1979) as a cell chaperone of simian virus 40 (SV40) large T antigen and was regarded as an oncoprotein of SV40 [1]. Subsequently, researchers found that p53 is not an oncoprotein but a tumor suppressor [2]. Mutations exist in p53 with more than 50% of tumors of various types [3,4]. Since then, p53 has been studied as the most frequent target for cancer research. p53 is a key cellular transcription factor that plays a central role in cellular responses to a broad range of stress factors through its regulation of a variety of cellular pathways such as apoptosis, cell cycle, cellular senescence, DNA repair, autophagy, and innate immune response [5].

It was firstly reported in 2003 that p53 could inhibit virus replication through enhancing apoptosis [6]. Since then, a growing number of studies on the relationship between viruses and p53 have been constantly reported. Based on these studies, viruses may target p53 in different modes. Some viruses like Zika virus (ZIKV) [7] and West Nile virus (WNV) [8] need active p53 to facilitate their replication, while others such as adenovirus [2], vaccinia virus [9], tanapox virus [10], and human papillomavirus (HPV) [11,12,13] inhibit p53 function during their infection. Interestingly, some viruses like SV40 [14], influenza A virus (IAV) [15,16,17], human immunodeficiency virus (HIV) [18], and herpes simplex virus (HSV) [19] act in a stage-specific dual manner regarding p53 regulation, activating p53 in one stage of the viral cycle and inhibiting it in the other stages [20]. In a word, regulatory roles of p53 during virus infection vary with specific viruses and even with infection stages, whereas the underlying mechanism remains to be elucidated.

Foot-and-mouth disease (FMD) is a highly contagious disease mainly infecting cloven-hoofed animals such as cattle, swine, and sheep [21,22]. It is endemic in many regions of the world and is responsible for significant economic losses [21]. Foot and mouth disease virus (FMDV), the causative agent of FMD, is a positive single-strand RNA virus belonging to the genus Aphthovirus of family Picornaviridae [23]. During FMDV infection, the host cytoplasmic virus sensors melanoma differentiation-associated gene 5 (MDA5) and retinoic acid-inducible gene-I (RIG-I) can perceive the viral RNA to trigger a signaling cascade, finally stimulating the expression of type-I interferon (IFN) and proinflammatory cytokines through nuclear transcription factor kappa B (NF-κB) and IFN-regulatory factors 3 and 7 (IRF 3/7) activation, thus establishing an antiviral state within infected cells [24,25,26]. Even though the main components of the innate immune signaling pathway against FMDV have been established, whether there are other proteins involved to regulate this pathway is largely unknown. Despite the well-established roles of p53 in innate immunity and the economic importance of FMD control, the exact roles of p53 during FMDV infection have not been documented so far. In this study, by using different cell lines and mice model, we convincingly demonstrated that p53 is required for efficient FMDV replication by suppressing innate immunity.

## 2. Materials and Methods

### 2.1. Cells and Virus

Baby hamster kidney (BHK-21) and porcine kidney (PK-15) cells were cultured in Dulbecco’s Modified Eagle Medium (DMEM) (Gibco, Grand Island, NY, USA), supplemented with 10% fetal bovine serum (FBS) (AusGeneX, Brisbane, Australia). Cells were incubated at 37 °C with 5% CO_2_. Foot-and-mouth disease virus type O/BY/2010 (FMDV O/BY2010) was cultured and propagated in BHK-21 cells. The 50% tissue culture infective doses (TCID_50_) were calculated according to the well-number with cytopathic effect (CPE) using the Reed and Muench method. All the virus-related experiments were conducted in the Animal Biological Safety Level 3 (ABSL-3) Laboratory of Lanzhou Veterinary Research Institute according to the protocol provided by the biosafety management committee.

### 2.2. Antibodies, Plasmids, and Chemical Treatment

VP1 rabbit polyclonal antibody was kindly provided by Dr. Haixue Zheng (Lanzhou Veterinary Research Institute). p53 (1C12) mouse monoclonal antibody (mAb) was purchased from Cell Signaling Technology (Beverly, MA, USA). β-actin mouse mAb was bought from Beijing Zhongshanjinqiao Biotechnology Co. Ltd. (Beijing, China). Primer synthesis and DNA sequencing were completed by Sangon Biotechnology Co. Ltd. (Shanghai, China). pSpCas9(BB) plasmid were provided by professor Feng Zhang (Broad Institute of Massachusetts Institute of Technology) [27]. HA-p53 and FLAG-p53 plasmids were respectively constructed in pCMV-HA and pCMV-3Tag-1 using enzyme digestion method. The primers used are listed in Supplementary Table S1. The proteasome inhibitor MG132 was purchased from Calbiochem (EMD Biosciences, San Diego, CA, USA). The p53 negative regulator murine double minute 2 (MDM2)-specific inhibitors NVP-CGM097 and Nutlin-3a were purchased from Selleck Chemicals (Houston, TX, USA). MG132, NVP-CGM097 and Nutlin-3a were all prepared in dimethyl sulfoxide (DMSO) as stock and were then added into medium to reach the final working concentration.

### 2.3. Virus Infection, RNA Extraction and RT-qPCR

For FMDV infection, monolayer of BHK-21, PK-15 cells and primary macrophages (Mφ), growing in logarithmic phase, were incubated at 37 °C with FMDV at the multiplicity of infection (MOI) of 0.1 for 1 h. After that, the virus solutions were aspirated, the cells were washed once and cultured in FBS-free DMEM. As a control, monolayer of these cells was cultured in the same volume of FBS-free DMEM. After treatment, the supernatant was removed and cells were washed three times with pre-chilled PBS and were then collected in TRIzol Reagent (Invitrogen, Carlsbad, CA, USA). Total RNA was extracted according to the manufacturer’s protocol (Tiangen Biotech, Beijing, China). cDNA diluted 20-fold was used as the template, and reverse transcription-quantitative real-time PCR (RT-qPCR) was performed on a CFX96 Real Time PCR System (Bio-Rad, Hercules, CA, USA). The relative expression of each gene was normalized with the reference gene (β-actin) by using the 2^−ΔΔCT^ calculation method. Differences were analyzed and graphed using the GraphPad Prism 5 software with the Student’s t test. The primers used are listed in Supplementary Table S1.

### 2.4. Western Blotting

Cells were lysed in the RIPA Lysis Buffer (Solarbio, Beijing, China). Total protein concentrations were determined using the BCA assay. Equal amounts of protein (40 μg) were separated by sodium dodecyl sulfate-polyacrylamide gel electrophoresis (SDS-PAGE) and the separated proteins were then transferred to polyvinylidene difluoride (PVDF) membrane (Millipore, Bedford, MA, USA). After that, the membrane was blocked with 5% skim milk at room temperature for 1 h, followed by incubation with primary p53 or VP1 antibody (1:1000 dilution) at 4 °C overnight, and then with horseradish peroxidase-conjugated secondary antibody at room temperature for 1 h. The targeted proteins were visualized using the Pierce chemiluminescence (ECL) substrate (Lot: TF268244, Thermo, Rockford, IL, USA).

### 2.5. Short Hairpin RNA (shRNA) Transfection

The shRNA method was used to downregulate p53 expression in BHK-21 cells. The plasmids used for expressing shRNA targeting p53 were designed and constructed by Shanghai Sangon Biotechnology Co. Ltd. (Shanghai, China). Briefly, three shRNAs targeting different regions of p53 exons, and a scrambled non-targeting shRNA (shCtrl) used as the negative control, were designed, synthesized, and ligated into the pSGU6/GFP/Neo vector. Transfections were performed using Lipofectamine 2000 reagent (Invitrogen, Carlsbad, CA, USA) following the manufacturer’s instructions. All the primers used for plasmid construction are shown in Supplementary Table S1.

### 2.6. Generation of p53 Knockout (KO) Cell Lines in BHK-21 and PK-15

p53-KO cell lines were established by using CRISPR/Cas9 system following the published protocols [14]. Briefly, two independent single guide RNAs (sgRNAs) targeting different positions of p53 were designed and ligated into the pSpCas9(BB) plasmid. The molecularly confirmed plasmids were transfected into BHK-21 or PK-15 cells and single cell clones were picked using cloning-ring anchoring method after puromycin selection and serial dilutions. The obtained cell clones were verified by sequencing and western blotting. The sequencing result of the p53-KO cell lines used in this study is shown in Supplementary Figure S1. The primers used for plasmid construction and sequencing are listed in Supplementary Table S1.

### 2.7. Reproduction and Identification of p53-KO Mice

Heterozygous p53-KO mice in C57BL/6 background were purchased from Beijing Biocytogen Co., Ltd. (Beijing, China) and were then maintained and reproduced under specific pathogen-free conditions in the Animal Center of Lanzhou Veterinary Research Institute. All the mice experiments were approved by the Animal Ethics Committee of Lanzhou Veterinary Research Institute, Chinese Academy of Agricultural Sciences (No. LVRIAEC2017-007, approved on 20/12/2017). The method for knocking out p53 in C57BL/6 mice was illustrated in Supplementary Figure S2. Briefly, p53 exons 2-7 of the targeted mouse were replaced with PGK-Neo cassette, resulting in the inactivation of p53. Reproduced descendants were genotyped with the PCR-based method illustrated in Supplementary Figure S2. The primers used for genotyping were provided by Beijing Biocytogen Co., Ltd. (Beijing, China); their sequences are listed in Supplementary Table S1.

### 2.8. Isolation and Culture of Primary Peritoneal Macrophages

Mice resident peritoneal macrophages (RPMφ) were collected following the method previously described [28]. Briefly, sterile normal saline solution (0.9% NaCl, *w/v*) was injected into the caudal half of the peritoneal cavity of the wild-type (WT) and p53-KO littermate mice using a 25-gage needle (Xi’an Jiaotong University, Shaanxi, China). The mouse body was gently kneaded and shaken for 15–20 s. Resident peritoneal cells were slowly withdrawn using a 19-gage needle (Xi’an Jiaotong University, Shanxi, China), and they were separated and purified by differential adhesion method. Non-adherent cells were removed by washing 3–5 times with warm PBS or FBS-free DMEM. Harvested RPMφ were seeded in 12-well plate and cultured in high-sugar DMEM (Gebico, MD, USA) supplemented with 20% FBS (AusGeneX, Brisbane, Australia).

### 2.9. Virus Challenge Assay in Mice

For in-vivo FMDV infection studies, four-week old and sex-matched WT and p53-KO littermate mice were intraperitoneally infected with FMDV (100 LD_50_). For the survival experiments, mice were monitored for survival after FMDV infection. For the virus load determination, mice were euthanized to death using cervical dislocation method and their hearts, spleens, and thigh muscles were sampled. RT-qPCR was used to quantify the relative level of viral RNA, and TCID_50_ method was used to determine the virus titer. For the histopathological diagnosis, the hearts, spleens, and thigh muscles from mock-treated or FMDV-infected mice were dissected, fixed in 4% paraformaldehyde solution, embedded into paraffin, sectioned, stained with hematoxylin-eosin (HE) solution, and examined by light microscopy. Histopathological experiments were accomplished by Chengdu Lilai Biotechnology (Chengdu, Sichuan, China). Data were calculated and graphed by GraphPad Prism 5 software.

## 3. Results

### 3.1. FMDV Infection Dynamically Regulates p53 Protein Level Partly through MDM2-Dependent Proteasome Pathway

**Figure 1:**
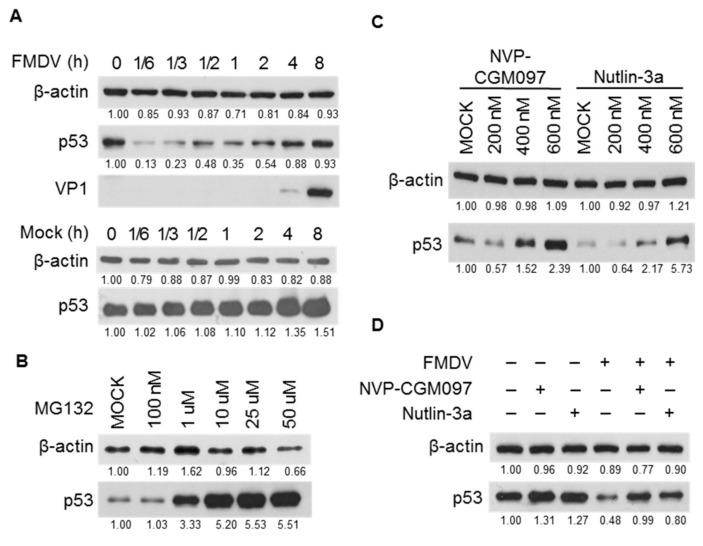
p53 protein level was dynamically regulated during foot-and-mouth disease virus (FMDV) infection partly through MDM2-dependent proteasome pathway. (**A**) p53 level was dynamically regulated during FMDV infection. BHK-21 cells, seeded in 60-mm dishes, were either mock-treated or infected with FMDV (multiplicity of infection (MOI) = 0.1) and harvested at the time points indicated. Western blotting (WB) was performed to detect p53 and VP1. (**B**) MG132 treatment increased the protein abundance of p53. BHK-21 cells were seeded in 6-well plates and were respectively treated with MG132 at a gradient concentration from 100 nM to 50 µM for 2 h. Samples were collected and then WB was performed to detect p53 expression. (**C**) Treatment with MDM2-specific inhibitor increased the p53 protein level. BHK-21 cells were plated in 60-mm dishes and then treated with MDM2 inhibitor NVP-CGM097 and Nutlin-3a at different concentrations (200 nM to 600 nM) for 12 h, and then the expression level of p53 was measured by WB. (**D**) Pre-treatment with MDM2 inhibitor compromised the reductive effect of FMDV infection on p53. BHK-21 cells were respectively pre-treated with 600 nM of the MDM2 inhibitor NVP-CGM097 and Nutlin-3a for 12 h. After that, cells were then either mock-treated or infected with FMDV (MOI = 0.1) for 30 min. Samples were harvested for WB to detect p53. The bands were quantified with ImageJ software and the relative expression value was given just below the corresponding band. All the experiments were repeated at least twice and similar results were obtained. β-actin was used as the internal control.

To investigate the effect of FMDV infection on p53 expression, p53 protein level was detected in BHK-21 cells after infection with FMDV for different time points. VP1 protein level was used to indicate the FMDV replication, which has been shown to be closely correlated with viral RNA replication (Figure S3). The results showed that FMDV infection caused the rapid reduction of p53 protein level as early as 1/6 h post infection (hpi). After 1 hpi, p53 protein level gradually increased and finally recovered to the basal level at 8 hpi when the viral VP1 protein was also obviously detected. In contrast, in the mock treatment group, p53 level was almost unchanged except for a slight increase at the later time points (Figure 1A). These data indicated that FMDV infection degrades p53 at the early stage of infection and the re-accumulation of p53 is correlated with FMDV replication at the later stage.

It is well known that proteins are degraded through three major protein degradation pathways in mammalian cells: proteasome, lysosome, and autophagosome [29]. It has been well-documented that the E3 ubiquitin ligase MDM2 serves as the p53 gatekeeper to promote the ubiquitination and subsequent proteasomal degradation of p53 [2]. To test whether the MDM2-dependent proteasome pathway is conserved in BHK-21 cells, the proteasome inhibitor MG132 and the MDM2-specific inhibitors NVP-CGM097 and Nutlin-3a were used. As shown in Figure 1B, MG132 treatment significantly increased the protein abundance of p53 in BHK-21 cells with a clear dosage effect, indicating proteasome pathway is involved in p53 turnover in this cell line. Consistently, treatment of BHK-21 cells with NVP-CGM097 or Nutlin-3a also promoted the accumulation of p53, which is also dosage-dependent (Figure 1C), indicating MDM2-dependent degradation of p53 is conserved in our cell line. Next, we tested whether MDM2 is involved in FMDV-induced rapid degradation of p53. As shown in Figure 1D, treatment with NVP-CGM097 or Nutlin-3a increased the p53 level and FMDV infection obviously reduced it as previously described. Importantly, pretreatment with either NVP-CGM097 or Nutlin-3a significantly compromised the reductive effect of FMDV infection on p53 level, strongly supporting that FMDV-induced degradation of p53 at the early infection stage is partly through MDM2-dependent proteasome pathway.

### 3.2. Depletion of p53 Inhibits FMDV Replication

**Figure 2:**
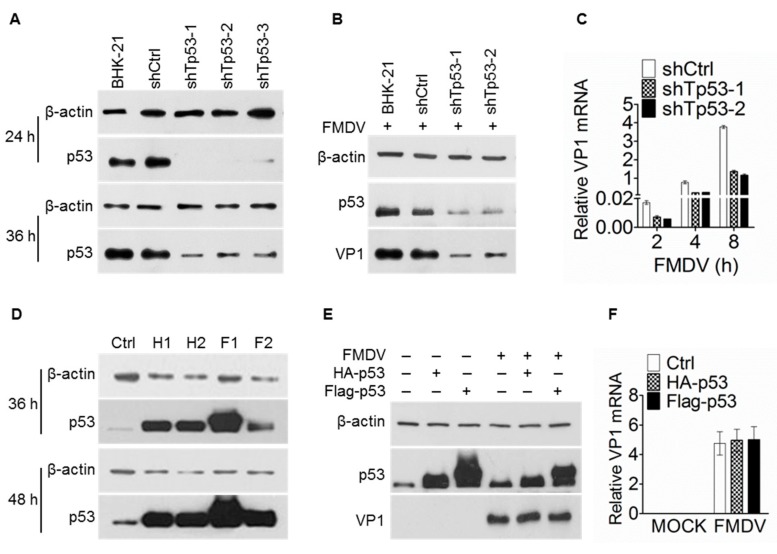
p53 knockdown reduced FMDV replication while its overexpression had no effect in BHK-21 cells. (**A**) Knockdown of p53 by short hairpin RNA (shRNA). BHK-21 cells were respectively transfected with plasmid expressing shRNA targeting p53 or a scramble shRNA as control (shCtrl). Cells were collected at 24 h and 36 h after transfection, and p53 level was detected by WB. (**B**) and (**C**) p53 knockdown reduced FMDV replication. BHK-21 cells were seeded in 6-well plates and were transfected with shRNA plasmid (1 and 2) to knockdown p53. At 24 h after transfection, Cells were infected with FMDV (MOI = 0.1) and collected at 8 h post infection (hpi) for (B) or at the indicated time points for (C). WB was used to detect the expression of VP1 and p53 at protein level in (B), while RT-qPCR was used to detect the relative level of VP1 mRNA in (C). (**D**) Optimization of p53 overexpression. BHK-21 cells were transfected with HA-p53 (H1 and H2), Flag-p53 (F1 and F2), or empty plasmid as control (Ctrl). p53 expression was detected by WB at 36 h and 48 h after transfection. (**E**) and (**F**) p53 overexpression had no significant effect on FMDV replication. BHK-21 cells were transfected with p53 overexpression plasmids (H1 and F1). At 48 h after transfection, cells were infected with FMDV (MOI = 0.1) for 8 h. WB (E) and RT-qPCR (F) were performed to detect the expression of VP1 at protein and mRNA level respectively. For A, B, D and E, the experiments were repeated at least twice and similar results were obtained. For C and F, the experiments were performed in triplicates and the bars are standard deviation (SD). β-actin was used as the internal control for both WB and RT-qPCR.

The dynamic regulation of p53 protein level upon FMDV challenge suggested the possible roles of this important protein during FMDV infection. In order to explore the effect of p53 on FMDV replication, three plasmids expressing short hairpin RNA (shRNA) targeting p53 were constructed and transfected into BHK-21 cell lines. As shown in Figure 2A, p53 protein was significantly downregulated after cells were transfected with shTp53 plasmids for 24 h and 36 h, with the reductive effect at 24 h after transfection more obvious, indicating the shRNA plasmids were successful and efficient. Therefore, BHK-21 cells were transfected with shTp53 plasmids for 24 h and then infected with FMDV (MOI = 0.1) for different time points. Western blotting and RT-qPCR were performed to detect the possible effects of p53 knockdown on FMDV replication. The results demonstrated that the expression of FMDV VP1 was significantly reduced at both the mRNA and protein levels in cells transfected with shTp53 plasmids (Figure 2B,C), indicating that p53 knockdown inhibited the FMDV replication.

Moreover, we also constructed p53-overexpressing plasmids (HA-p53 and Flag-p53) to test the effects of p53 overexpression on FMDV replication. As shown in Figure 2D, p53 protein level is obviously up-regulated at both 36 h and 48 h after cells were transfected with HA-p53 (H1 and H2) or Flag-p53 (F1 and F2) plasmids, indicating the overexpression was successful. However, the VP1 protein level and RNA level were not significantly changed when p53 was obviously overexpressed (Figure 2E,F), demonstrating that overexpression of p53 does not have any significant effect on FMDV replication. These data suggested that only basal levels of p53 are efficient in regulating FMDV replication.

**Figure 3:**
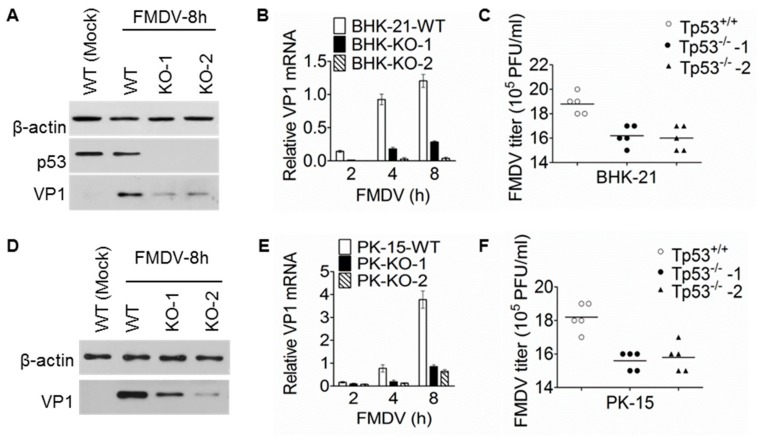
p53 knockout inhibited FMDV replication in BHK-21 and PK-15 cells. (**A**–**C**). p53 knockout inhibited FMDV replication in BHK-21 cells. Wild-type (WT) BHK-21 and the verified p53 knockout (KO) cells were seeded in 60-mm plates. Cells were either mock-treated or infected with FMDV (MOI = 0.1). At the indicated time points, samples were collected for WB (A) and RT-qPCR (B) to detect VP1 at protein and mRNA level respectively. For (C), different cell lines were seeded in T25 cell culture flask and were then infected with 1ml of FMDV (TCID^50^ = 10^−6^). After serial passages, virus titer was determined using TCID_50_ method at the 5th passage. (**D**–**F**) p53 knockout inhibited FMDV replication in PK-15 cells. The experiments were performed exactly the same as in (A–C), except the cell lines used were in PK-15 background. (D) is the WB result showing VP1 protein level, (E) is the RT-qPCR result showing VP1 mRNA level, and (F) shows virus titer at the 5th passage. For A and D, the experiments were performed at least twice and similar results were obtained. For B and E, the experiments were performed in triplicates and the bars are SD. β-actin was used as the internal control for both WB and RT-qPCR. For C and F, the virus titer was determined with 5 replicates, and the lines represent average values.

CRISPR/cas9 system is a newly developed technique and is now widely used for genome editing in mammalian cells [27]. To unbiasedly confirm the effect of p53 depletion on FMDV replication, we also used this technique to knockout p53 in BHK-21 and PK-15 cell lines. Two sgRNAs targeting the separate sites of p53 exons were respectively designed for BHK-21 and PK-15 cells. Single cell clones were isolated and propagated after cells were transfected with CRISPR plasmids. As shown in Supplementary Figure S1, two independent cell clones with different mutation sites were successfully identified in both BHK-21 and PK-15 cells, and the out-of-frame mutation caused the pre-mature translational stop and the truncated p53 proteins, indicating the p53 knockout was successful. The knock-out of p53 in BHK-21 cells was also confirmed with western blotting (Figure 3A), but unluckily the p53 antibody we used can’t detect the specific band in PK-15 cells. Hence, we used these p53-KO cell lines to perform FMDV infection assay. As shown in Figure 3A–C, VP1 protein level, VP1 RNA level, and also the FMDV titer were obviously reduced in the p53-KO BHK-21 cell lines after FMDV infection. Similar results were also obtained in the p53-KO PK-15 cell lines (Figure 3D–F). Together, these data are consistent with those from the p53 knockdown experiments, unequivocally demonstrating that basal level p53 is required for efficient FMDV replication.

### 3.3. Expression of the Immune-Related Genes is Greatly Enhanced in p53-KO Cells

**Figure 4:**
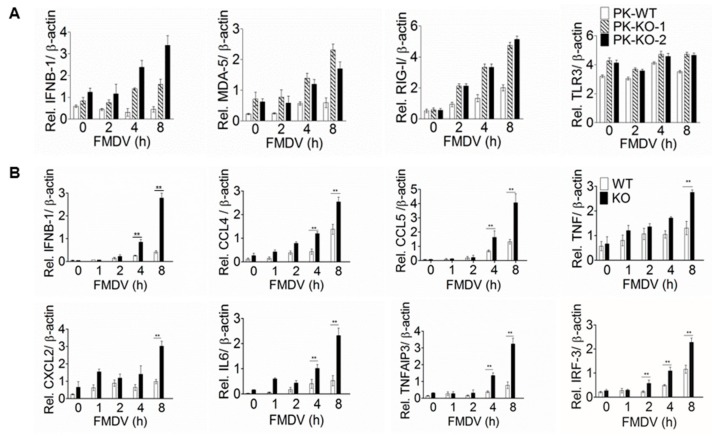
Knockout of p53 enhanced the expression of immune-related genes. (**A**) Knockout of p53 in PK-15 enhanced the expression of IFNB-1 and virus sensors upon FMDV infection. WT PK-15 and two p53 knockout cell lines were seeded in 60-mm dishes and were then infected with FMDV (MOI = 0.1). At the indicated time points, cells were collected for RT-qPCR to detect the expression level of IFNB-1, MDA-5, RIG-I and TLR3. (**B**) Expression of IFNB-1 and several cytokines was significantly increased in the primary peritoneal macrophages isolated from p53-KO mice. Primary peritoneal macrophages were respectively isolated from WT and p53-KO mice and cultured in 12-well plates. Cells were then infected with FMDV (MOI = 0.1) and harvested at the indicated time points. RT-qPCR was used to detect the relative expression of IFNB-1 and the other cytokines. The experiments were performed in triplicates and the bars are SD. ** indicates significant differences at *p* < 0.01 according to Student’s t test. β-actin was used as the internal control.

Recognition of viral nucleic acids with pattern recognition receptors (PRRs) is the first step to induce innate immune system [30]. Many RNA viruses replicate in the cytoplasm where they are sensed by the cytoplasmic TLRs, MDA5, and RIG-I. In a typical scenario, the viral RNA can be perceived by these PRRs to trigger the expression and release of interferons causing nearby cells to strengthen their antiviral response. It has been well-documented that p53 is involved in innate immune regulation through modulating the expression of the type-I IFN and the induction of various cytokines [31,32]. To investigate the effects of p53 on the innate immune response, we detected the relative expression level of IFNB-1 and the host sensors MDA5, RIG-I, and TLR3 in the p53-KO PK-15 cells after FMDV challenge. The results showed that after FMDV infection the expression level of IFNB-1, MDA5 and RIG-I in the p53-KO cells was clearly higher than that in the WT, while there are no obvious changes for TLR3 (Figure 4A).

p53 was shown to influence the innate immune system by modulating macrophage (Mφ) function [33]. p53 deficiency in macrophages enhances the production of pro-inflammatory cytokines such as IL-1, IL-6, IL-12, and TNF [34]. Our pre-test experiment demonstrated that FMDV can infect the primary resident peritoneal macrophages (RPMφ) isolated from the C57BL/6 mice without the assistance of specific antibodies and shows restricted replication in those cells (Supplementary Figure S4). In order to investigate the effects of p53 on FMDV-induced cytokines in macrophages, RPMφ were respectively isolated from the WT and p53-KO mice and they were infected with FMDV for different time. RT-qPCR was performed to detect the expression of immune-related cytokines. As shown in Figure 4B, compared with WT Mφ, the induction of cytokines, such as CCL4, CCL5, CXCL2, IL6, IFNB-1, TNFAIP3, and IRF3, were significantly enhanced in p53-KO Mφ. Collectively, these data confirmed that knockout of p53 enhanced the expression of immune-related genes, including the FMDV sensors MDA5 and RIG-I, IFNB-1, and several cytokines.

### 3.4. p53-KO Mice is More Resistant to FMDV Infection

**Figure 5:**
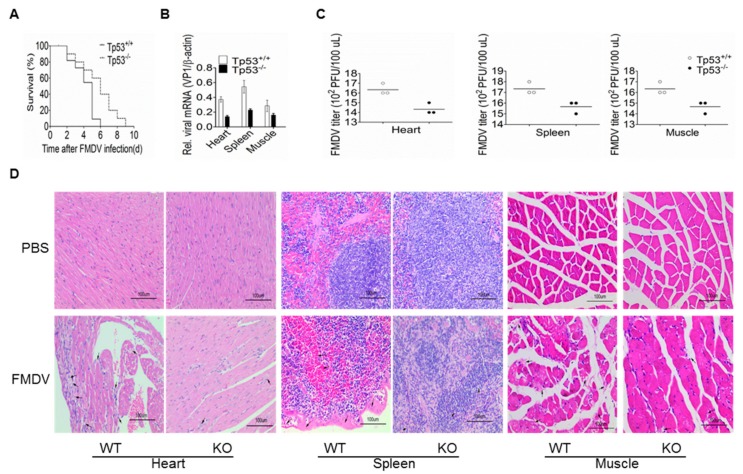
Effects of p53 knockout on FMDV replication and pathogenicity in mice. (**A**) FMDV-induced death was delayed in p53-KO mice. Four-week-old WT and p53-KO mice were intraperitoneally injected with FMDV (100 LD_50_), and the survival rate was calculated each day. The experiment was repeated twice and a similar result was obtained. *n* = 10 for each experiment. (**B**) FMDV RNA was reduced in the hearts, spleens and muscles of p53-KO mice. The adult mice were challenged with FMDV using the same method as described in (A). Mice were euthanized at 3 days post-infection (dpi) and their hearts, spleens, and thigh muscles were sampled for RNA extraction. RT-qPCR was performed to quantify the relative level of VP1 mRNA. The experiment was performed in triplicates and the bars are SD. (**C**) FMDV titer was reduced in the hearts, spleens, and muscles of p53-KO mice. Samples were collected the same as in (B), and the virus titer was determined using TCID_50_ method. *n* = 3. The lines represent average values. (**D**) Histopathological changes in the hearts, spleens, and muscles of p53-KO mice were alleviated. Samples were collected the same as in (B) and were immediately fixed with 4% paraformaldehyde solution for further sectioning and hematoxylin and eosin (HE) staining. Note the black arrows indicate abnormal histopathological changes. Three biological replicates were taken for each treatment group and the representative images were shown. Bars = 100 μm.

To investigate the effects of p53 on FMDV infection and replication in-vivo, WT and p53-KO mice were intraperitoneally infected with FMDV, and then the mice survival rate and the virus load were compared. As shown in Figure 5A, all of the WT mice died within 6 days, while the p53-KO mice showed a delayed death, surviving until 9 days after infection. RT-qPCR and TCID_50_ assay were also performed to detect the VP1 expression and virus titer. Results showed that compared to WT mice, the VP1 expression and virus titer were reduced in the heart, spleen, and muscle of p53-KO mice (Figure 5B,C). These results demonstrated that knockout of p53 inhibited FMDV replication in-vivo.

Based on our findings that virus load was reduced in the heart, spleen, and muscle of p53-KO mice, histopathological diagnosis was then performed in these organs to check for possible changes. After sectioning and HE staining, the microscopic results showed that abnormal pathological changes can be clearly observed in the WT mice. In the heart, some cardiomyocytes degenerated, showing necrotic features, some broken myocardial fibers, and lymphocytes infiltrated in myocardial fibers and interstitium, but all these phenomenon were alleviated in the p53-KO mice. In the spleen, reduction and necrosis of spleen lymphocytes appeared in both the WT and p53-KO group, which means lymphocytes were phagocytized by phagocytic cells, resulting in impaired immunity. However, compared with p53-KO mice, in WT group, the splenic capsule shrunk more severely, the red pulp expanded and the white pulp atrophied, the splenic corpuscles in the white pulp looked smaller, more red blood cells could be seen in the spleen sinus, and more lymphocytes necrosed in the white pulp, indicating that FMDV infection had caused more severe damage to the spleens in the WT group. In the muscles from the WT mice, there were more areas with indistinct cell margins and incomplete cell morphology (Figure 5D). Together, these results demonstrated that p53-KO mice are more resistant to the FMDV-induced pathogenesis.

## 4. Discussion

### 4.1. p53 is Required for Efficient FMDV Replication

p53 is a transcription factor and major tumor suppressor that plays a major role in regulating cellular responses to DNA damage and other genomic aberrations. Activation of p53 can lead to either cell cycle arrest and DNA repair or apoptosis [35]. Since it was initially reported that p53 has antiviral function against Vesicular Stomatitis Virus (VSV) and Newcastle disease virus (NDV) in 2003 [6], a growing number of studies about the relationship between p53 and viruses are now being reported [20]. It can be clearly seen from these studies that whether p53 plays positive or negative roles in virus replication varies with specific virus-host interaction. A positive example is ZIKV, whose capsid protein can bind to p53 E3 ligase MDM2 and thus prevent the formation of the MDM2-p53 complex and the degradation of p53. The elevated p53 triggers the apoptosis of neural cells, which is most likely the core for ZIKV-induced neuronal defects and congenital microcephaly [36]. Another contrast example is HPV, which takes advantage of its viral protein E6 that inhibits the p53 activity to induce viral replication and lead to malignant transformation [11]. Here we provide convincing data to demonstrate that p53 is required for efficient FMDV replication. First, knockdown of p53 in BHK-21 cells significantly reduced the mRNA abundance and protein level of VP1 after FMDV infection (Figure 2B,C). Second, FMDV replication was significantly inhibited in the p53 knock-out cell lines both in BHK-21 and PK-15 background, shown by western blotting, RT-qPCR and also virus titer determination (Figure 3). And last, viral challenge assay with p53 knock-out mice proved that the viral load in heart, spleen, and muscle was decreased, FMDV-induced histopathological changes were alleviated, and the lifespan was extended in the p53 knockout mice (Figure 5). Together, these data proved that depletion of p53 in both cell lines and mice reduced FMDV replication. However, overexpression of p53 in BHK-21 cells didn’t have any significant effect on FMDV replication (Figure 2D–F), indicating only basal level p53 is important to facilitate FMDV replication. It is possible that p53 needs other co-factors to exert this function. Hence, overexpression of p53 alone can’t see any significant effect.

### 4.2. p53 Suppresses the Innate Immune Response During FMDV Infection

Upon viral infection, the major defensive strategy employed by the host immune system is the induction of type I IFN, which promotes the expression of IFN-stimulated genes (ISGs) to act antiviral function [30]. Consistent with its contrasting effects on the replication of different viruses, p53 can also regulate the innate immune signaling differently depending on the specific virus-host context. On the one hand, p53 contributes to immune responses by directly activating key regulators of immune signaling pathways, including IRF5 [37], ISG15 [38], IRF9 [39], and RIG-I [40]. Moreover, transcription of the p53 gene is induced by IFN signaling, suggesting a positive feedback loop involving p53 to enhance type I IFN signals [6]. Indeed, it was reported that knockdown of p53 expression by RNAi enhanced IAV replication and reduced the expression of immune-related genes, such as IRF7, IRF9, ISG15, ISG20, GBP1, RIG1, and OAS1 [16]. In contrast, in some other studies, p53 has also been reported to inhibit signal transducer and activation of transcription 1 (STAT1), a transcription factor that drives the expression of ISGs and pro-inflammatory cytokines. In addition, the promoter of IL6 and NF-κB can also be directly repressed by p53 [32,41,42]. Hence, the available studies indicate that p53 can act as either a transcriptional activator or a transcriptional repressor [2]. Our study supported that p53 suppresses the expression of immune-responsive genes upon FMDV replication, at least in PK-15 cells and C57BL/6 mice. After FMDV replication, the expression of IFNB-1, MDA-5, and RIG-I was obviously enhanced in p53 KO PK-15 cells (Figure 4A). Consistently, in the primary peritoneal macrophages isolated from p53 KO mice, the expression level of IFNB-1 and several cytokines was also significantly increased compared to those from WT mice (Figure 4B). In contrast to our conclusion, a recent paper involving the p53-mediated innate immunity during FMDV infection described that a chemical compound 5-Fluorouracil (5-AU) widely used to induce cellular p53 stabilization can induce the expression of ISG20, IRF9, RIG-I and ISG15, but these experiments were performed in HEK-293T cells that FMDV can’t infect [43]. Considering that the roles of p53 in regulating innate immunity strictly depends on the specific context of virus-host cell pair as discussed above and our data are obtained from the more natural contexts, this seemingly contradictory conclusion can be then understood. It will be interesting in the future to investigate the underlying molecular mechanism of the differential regulation of innate immunity by p53 in different context. One possibility is that targeting some of the immune signaling genes may be relatively conserved, but either stimulatory or suppressive effect depends on the presence of functional coactivators or co-suppressors in specific context.

Being an important regulator of host innate immune signaling, p53 can affect the replication efficiency of many viruses. Therefore, it is not surprising that p53 is a prominent candidate for viral targets. During the infection of different viruses or even at the different infection stages of the same virus, the protein level or activity of p53 can be differentially modulated [20]. Here, we showed that p53 protein level was dynamically regulated during FMDV infection. At the early infection stage, p53 was rapidly degraded partly through MDM2-dependent mechanism (Figure 1). Considering the conclusion that p53 suppresses the innate immunity and knock-out of p53 inhibits FMDV replication in our system, degradation of p53 at the early stage should be a host antiviral immune response triggered by FMDV infection. In this way, the p53 gatekeeper MDM2 acts more like a FMDV sensor to degrade the basal level p53 and enhance the host immune response. However, how FMDV infection was rapidly sensed by MDM2 still remains elusive. At the mid and late stage of infection, the reduced p53 was gradually increased and finally recovered back to the basal level, indicating the degradation of p53 is inhibited. Thinking about p53 is required for efficient FMDV replication, and this effect might be an immunosuppressive effect incurred by some FMDV proteins. Interacting with MDM2 and thus disrupting the formation of MDM2-p53 complex seems to be a widely used strategy by many viral proteins to elevate p53 level, such as ZIKV capsid protein [36], WNV capsid protein [8], and IAV nucleoprotein (NP) [44]. Hence, it would be interesting to test whether some of the FMDV proteins directly interact with MDM2. In short, presumably, the dynamic regulation of p53 level during FMDV replication is a reflection of tense rivalry between virus and host cell: rapid degradation at the early stage to enhance the immune response, whereas the recovery at the late stage is possibly incurred by FMDV proteins to suppress immunity and facilitate viral replication.

## Supplementary Materials

The following are available online at https://www.mdpi.com/1999-4915/11/8/727/s1, Figure S1: Characterization of p53 knockout (KO) cell lines, Figure S2: Genotyping of p53 knockout (KO) mice, Figure S3: Correlation of VP1 protein level with FMDV replication, Figure S4: FMDV infects mice peritoneal macrophages, Table S1: List of the primers used in this study.
