# Supplementary material for: Basal Level p53 Suppresses Antiviral Immunity Against Foot-And-Mouth Disease Virus
Source: Viruses. 2019 Aug 7;11(8):727. doi: 10.3390/v11080727 (PMC6723088; doi:10.3390/v11080727)
Supplement: Supplementary file 1 [file viruses-11-00727-s001.zip › viruses-545305-for conversion-supplementary/Supplementary Materials/Supplemental figures.docx]

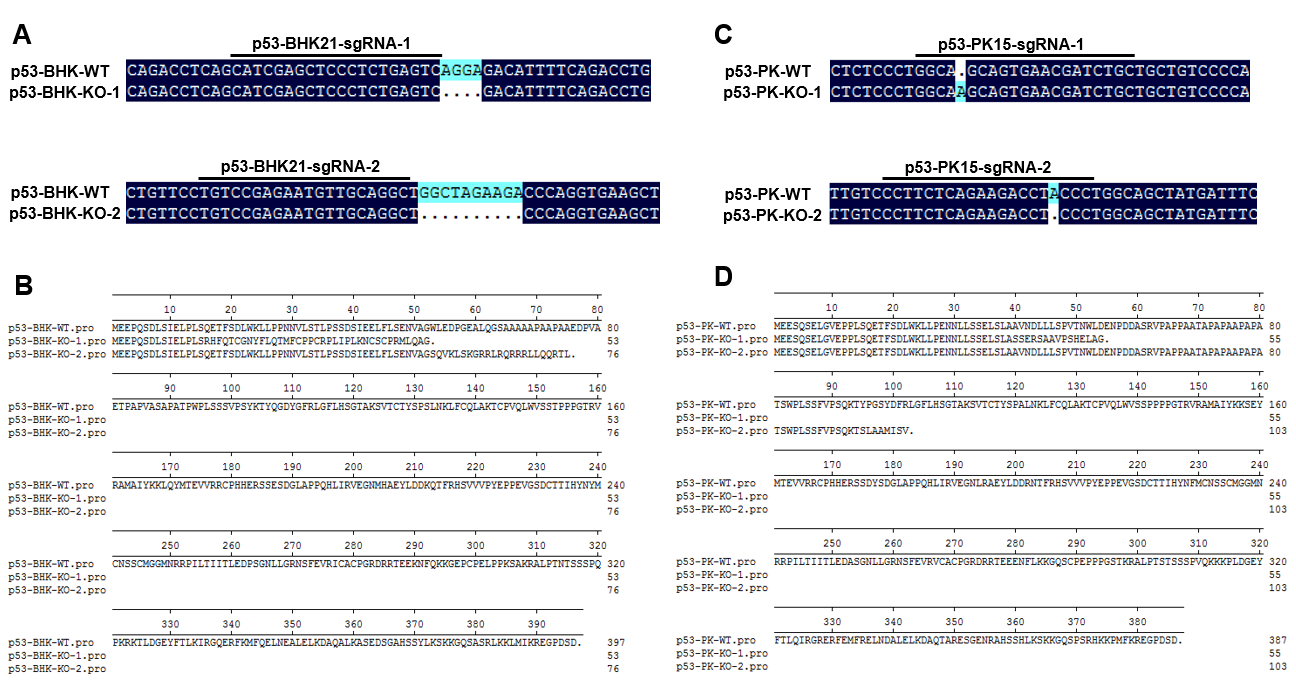


**Supplementary Figure S1. Characterization of p53 knockout (KO) cell lines.**

**(A)** Partial alignment of the DNA sequences spanning the mutation site amplified from the wild-type (WT) and p53 KO cell lines in BHK-21. **(B)** Alignment of the predicted p53 protein sequences of the WT and p53 KO cell line in BHK-21. **(C)** Partial alignment of the DNA sequences spanning the mutation site amplified from the WT and p53 KO cell lines in PK-15. **(D)** Alignment of the predicted p53 protein sequences of the WT and p53 KO cell line in PK-15. Noting that the out-of-frame mutation in the p53 KO cell lines cause the truncated p53 proteins.


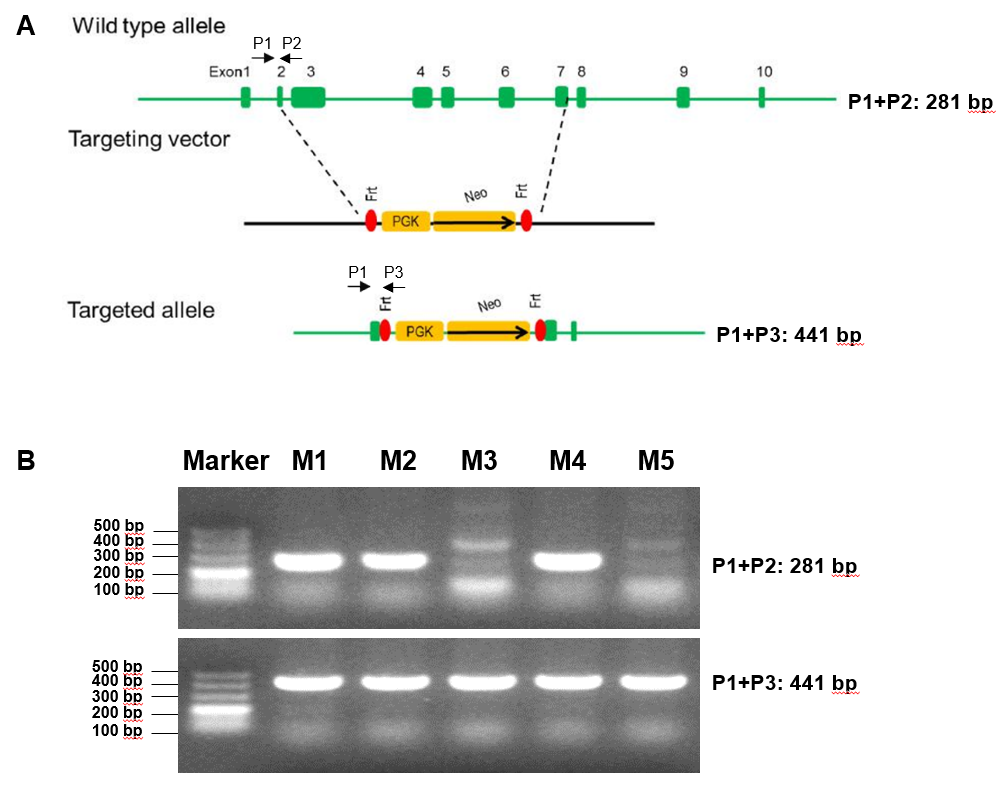


**Supplementary Figure S2. Genotyping of p53 knockout (KO) mice.**

**(A)** A schematic graph showing the generation of p53 KO mice. P1, P2 and P3 are the primers used for genotyping. **(B)** A representative gel image showing the genotyping result of p53 KO mice. Noting that M3 and M5 are Tp53^-/-^ homozygous mice, while the others are Tp53^+/-^ heterozygous mice.


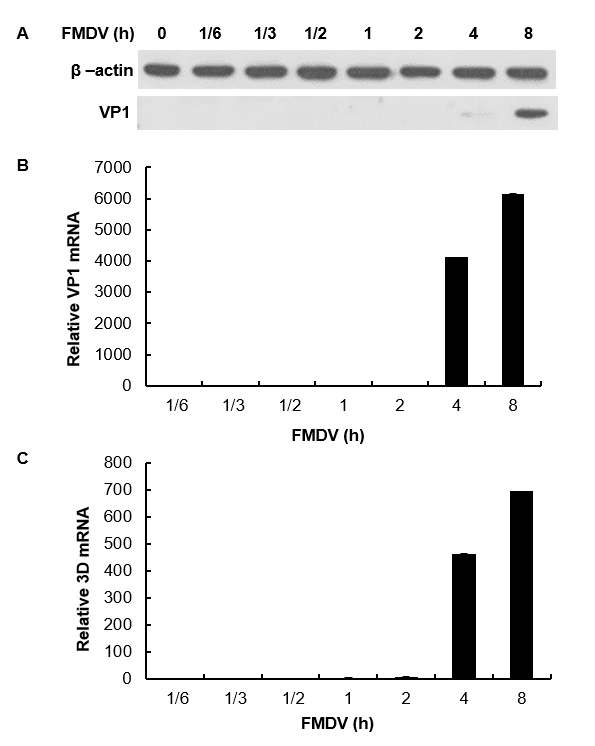


**Supplementary Figure S3. Correlation of VP1 protein level with FMDV replication.**

**(A)** Western blot result showing the accumulation of VP1 protein at the different time points after FMDV infection (0.1 MOI) in BHK-21 cells. **(B)** and **(C)** RT-qPCR results showing the FMDV replication in BHK-21 cells at the different time points after FMDV infection (0.1 MOI), indicated by relative VP1 mRNA **(B)** and nonstructural 3D mRNA **(C)**.


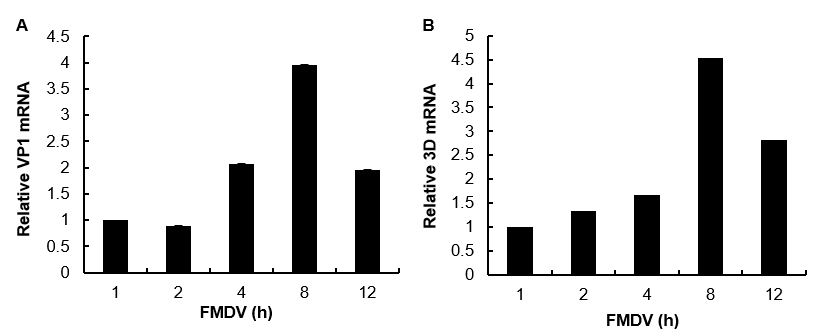


**Supplementary Figure S4. FMDV infects mice peritoneal macrophages.**

**(A)** and **(B)** RT-qPCR results demonstrating that FMDV infects mice peritoneal macrophages and shows restricted replication in these cells, indicated by the accumulation of VP1 mRNA **(A)** and nonstructural 3D mRNA **(B)** at the different time points after FMDV infection (0.1 MOI).
