# Supplementary material for: Basal Level p53 Suppresses Antiviral Immunity Against Foot-And-Mouth Disease Virus
Source: Viruses. 2019 Aug 7;11(8):727. doi: 10.3390/v11080727 (PMC6723088; doi:10.3390/v11080727)
Supplement: Supplementary file 1 [file viruses-11-00727-s001.zip › viruses-545305-for conversion-supplementary/Supplementary Materials/Supplemental Figures.pptx]

## Slide 1
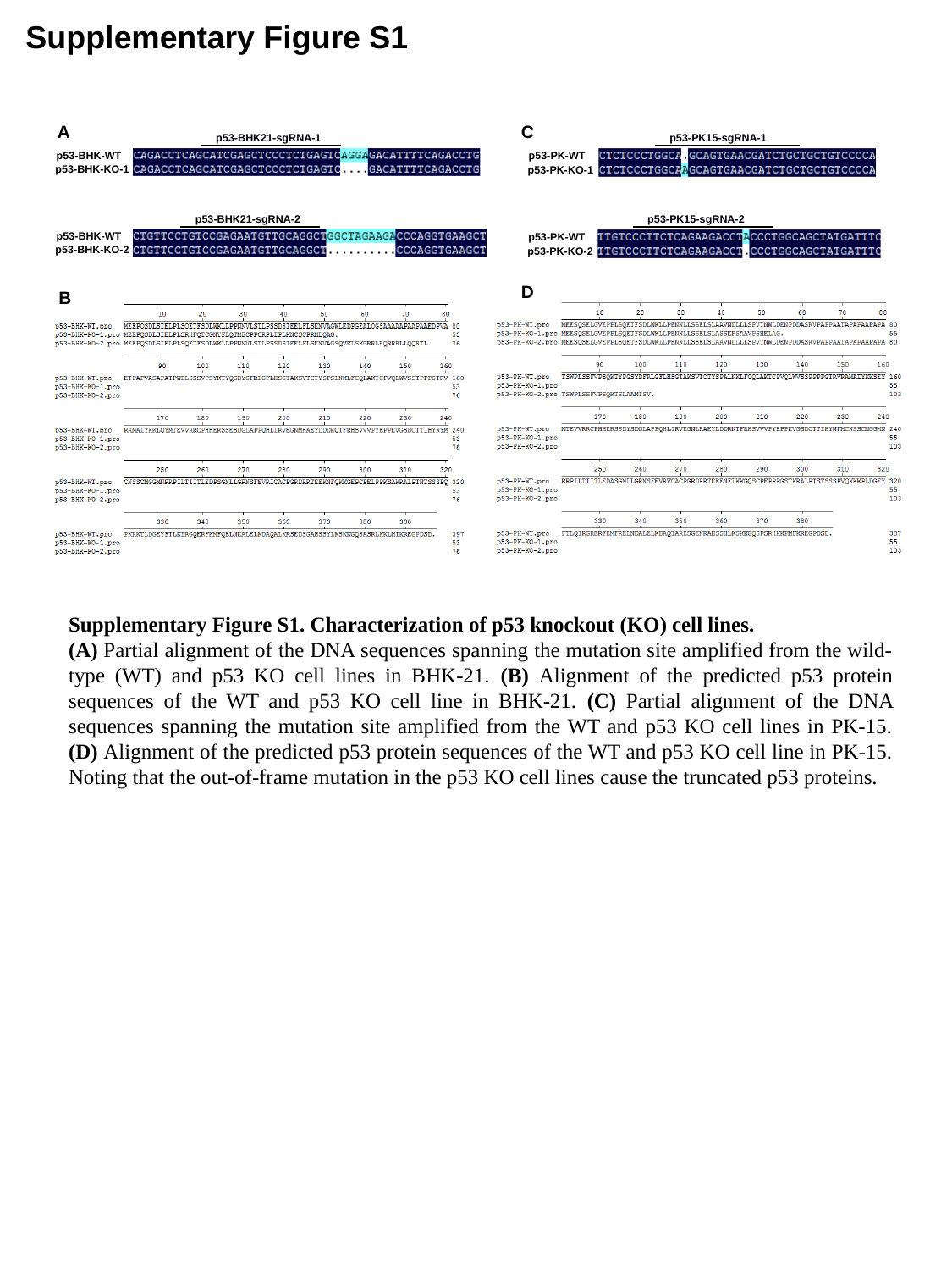

Supplementary Figure S1
A
C
p53-BHK21-sgRNA-1
p53-BHK-WT
p53-BHK-KO-1
p53-BHK21-sgRNA-2
p53-BHK-WT
p53-BHK-KO-2
p53-PK15-sgRNA-1
p53-PK-WT
p53-PK-KO-1
p53-PK15-sgRNA-2
p53-PK-WT
p53-PK-KO-2
D
B
Supplementary Figure S1. Characterization of p53 knockout (KO) cell lines.
(A) Partial alignment of the DNA sequences spanning the mutation site amplified from the wild-type (WT) and p53 KO cell lines in BHK-21. (B) Alignment of the predicted p53 protein sequences of the WT and p53 KO cell line in BHK-21. (C) Partial alignment of the DNA sequences spanning the mutation site amplified from the WT and p53 KO cell lines in PK-15. (D) Alignment of the predicted p53 protein sequences of the WT and p53 KO cell line in PK-15. Noting that the out-of-frame mutation in the p53 KO cell lines cause the truncated p53 proteins.

## Slide 2
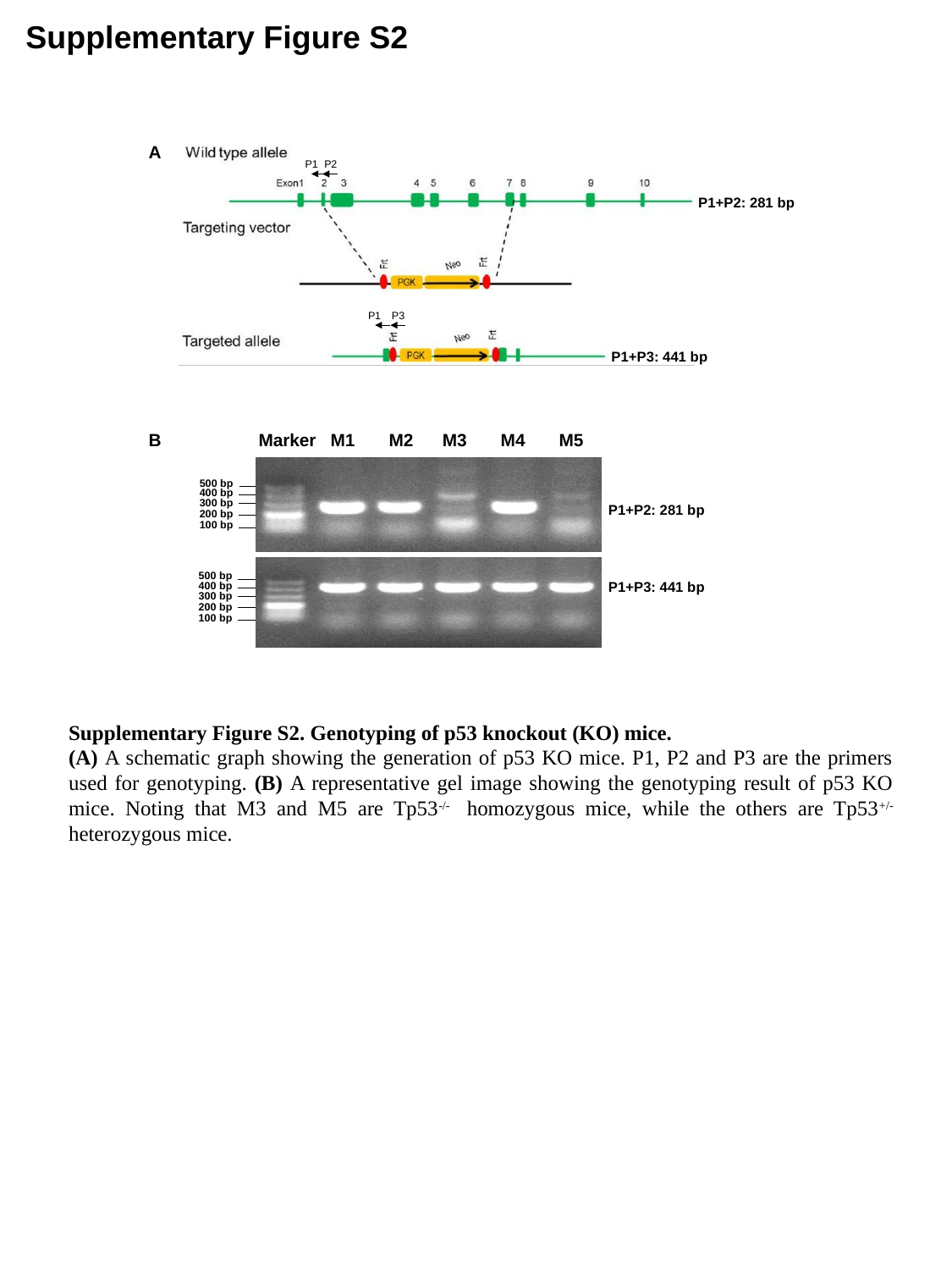

Supplementary Figure S2
A
P1
P2
P1+P2: 281 bp
P1
P3
P1+P3: 441 bp
B
Marker M1 M2 M3 M4 M5
500 bp
400 bp
300 bp
P1+P2: 281 bp
200 bp
100 bp
500 bp
P1+P3: 441 bp
400 bp
300 bp
200 bp
100 bp
Supplementary Figure S2. Genotyping of p53 knockout (KO) mice.
(A) A schematic graph showing the generation of p53 KO mice. P1, P2 and P3 are the primers used for genotyping. (B) A representative gel image showing the genotyping result of p53 KO mice. Noting that M3 and M5 are Tp53-/- homozygous mice, while the others are Tp53+/- heterozygous mice.

## Slide 3
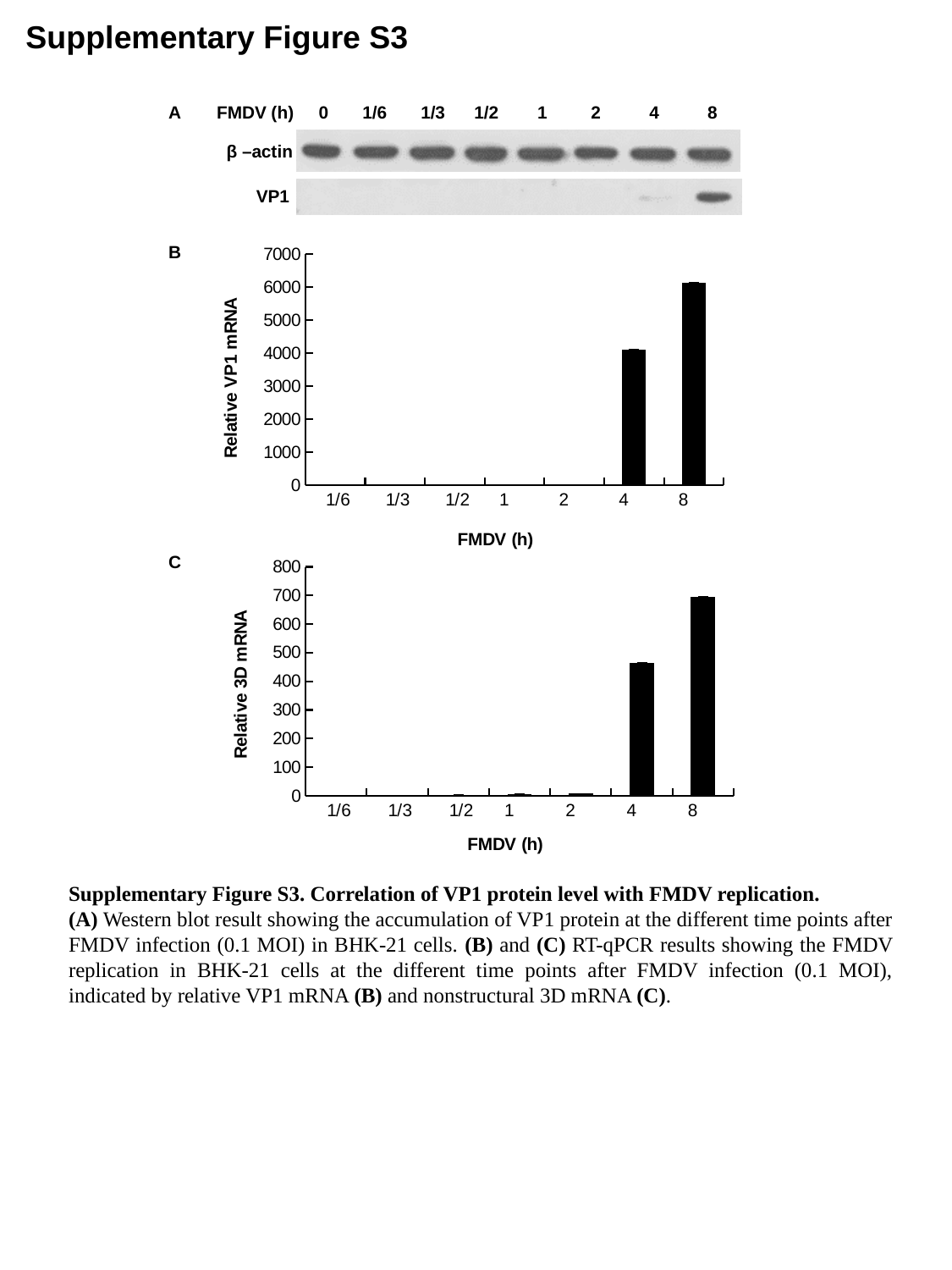

Supplementary Figure S3
A
FMDV (h)
 0 1/6 1/3 1/2 1 2 4 8
β –actin
VP1
B
### Chart
| Category | VP1 |
|---|---|
| 0.16666666666666666 | 1.0 |
| 0.33333333333333331 | 2.228338753553735 |
| 0.5 | 4.530735233690392 |
| 1 | 7.234662386887021 |
| 2 | 17.245676483271698 |
| 4 | 4118.192931276353 |
| 8 | 6147.38104812923 |C
### Chart
| Category | VP1 |
|---|---|
| 0.16666666666666666 | 1.0 |
| 0.33333333333333331 | 1.3282203502151912 |
| 0.5 | 2.8332744411460435 |
| 1 | 5.716572981897799 |
| 2 | 7.806612359260257 |
| 4 | 464.1632490677864 |
| 8 | 695.0100451582787 |Supplementary Figure S3. Correlation of VP1 protein level with FMDV replication.
(A) Western blot result showing the accumulation of VP1 protein at the different time points after FMDV infection (0.1 MOI) in BHK-21 cells. (B) and (C) RT-qPCR results showing the FMDV replication in BHK-21 cells at the different time points after FMDV infection (0.1 MOI), indicated by relative VP1 mRNA (B) and nonstructural 3D mRNA (C).

## Slide 4
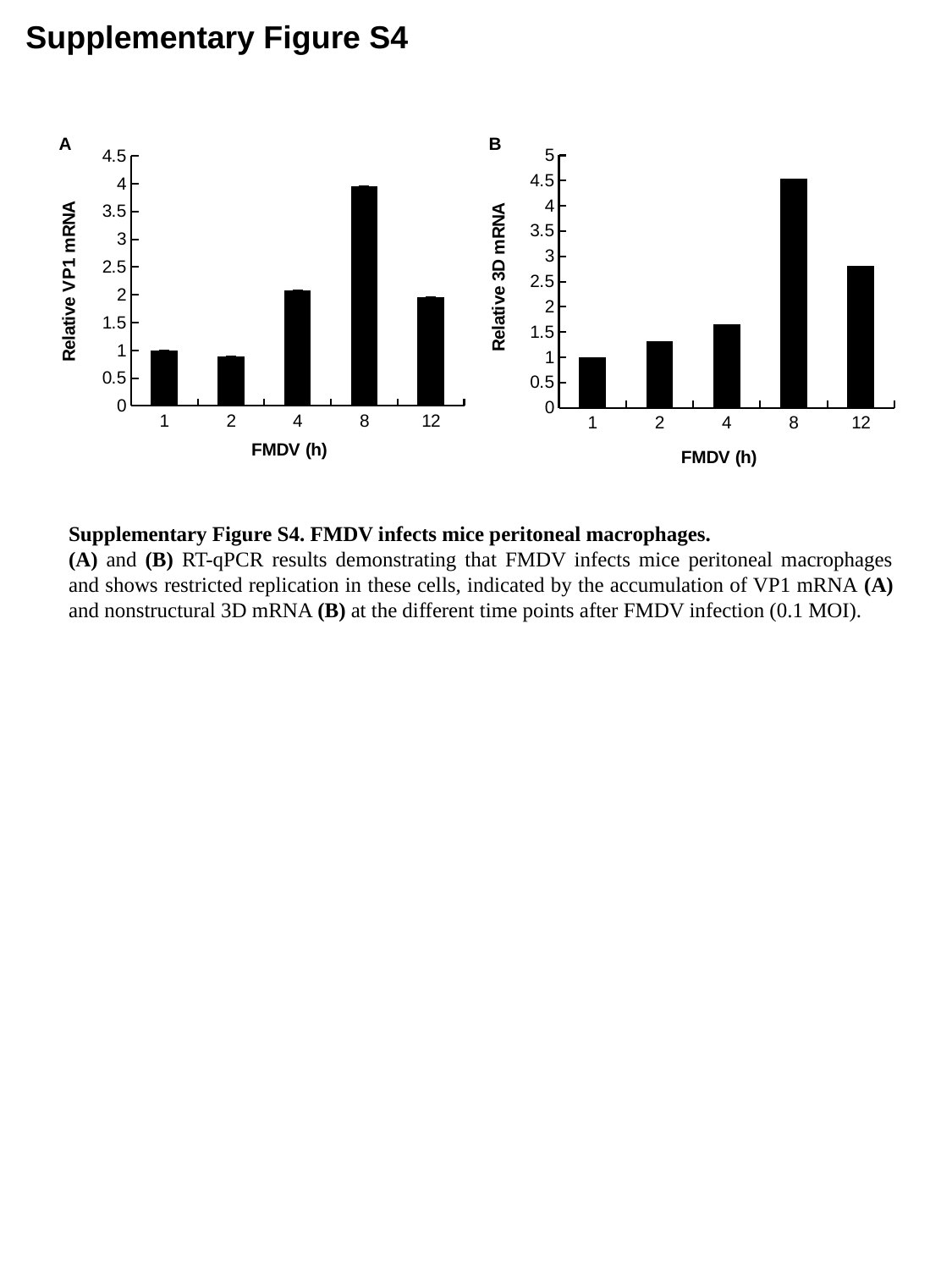

Supplementary Figure S4
A
B
### Chart
| Category | VP1 |
|---|---|
| 1 | 1.0 |
| 2 | 0.892711 |
| 4 | 2.0744940449373996 |
| 8 | 3.957297628068606 |
| 12 | 1.9621803277950935 |
### Chart
| Category | VP1 |
|---|---|
| 1 | 1.0 |
| 2 | 1.3219597302384307 |
| 4 | 1.6595584863558632 |
| 8 | 4.542502918021365 |
| 12 | 2.809948598333171 |Supplementary Figure S4. FMDV infects mice peritoneal macrophages.
(A) and (B) RT-qPCR results demonstrating that FMDV infects mice peritoneal macrophages and shows restricted replication in these cells, indicated by the accumulation of VP1 mRNA (A) and nonstructural 3D mRNA (B) at the different time points after FMDV infection (0.1 MOI).
