# Supplementary material for: Basal Level p53 Suppresses Antiviral Immunity Against Foot-And-Mouth Disease Virus
Source: Viruses. 2019 Aug 7;11(8):727. doi: 10.3390/v11080727 (PMC6723088; doi:10.3390/v11080727)
Supplement: Supplementary file 1 [file viruses-11-00727-s001.zip › viruses-545305-for conversion-supplementary/Supplementary Materials/Supplemental Table 1.docx]

**Supplementary Table S1. List of the primers used in this study**

| **Primer names** | **Sequences** | |
| --- | --- | --- |
| shRNA-NC | **F**: CACCGTTCTCCGAACGTGTCACGTCAAGAGATTACGTGACACGTTCGGAGAATTTTTTG  **R**: AGCTCAAAAAATTCTCCGAACGTGTCACGTAATCTCTTGACGTGACACGTTCGGAGAAC | |
| shRNA-1 | **F**: CACCGCTCCTCCTCAGCATCTTATCTTCAAGAGAGATAAGATGCTGAGGAGGAGCTTTTTTG  **R**: AGCTCAAAAAAGCTCCTCCTCAGCATCTTATCTCTCTTGAAGATAAGATGCTGAGGAGGAGC | |
| shRNA-2 | **F**: CACCGGAAATATGCATGCCGAATACTTCAAGAGAGTATTCGGCATGCATATTTCCTTTTTTG  **R**: AGCTCAAAAAAGGAAATATGCATGCCGAATACTCTCTTGAAGTATTCGGCATGCATATTTCC | |
| shRNA-3 | **F**: CACCGCTAAACGAGCATTGCCTACCTTCAAGAGAGGTAGGCAATGCTCGTTTAGCTTTTTTG  **R**: AGCTCAAAAAAGCTAAACGAGCATTGCCTACCTCTCTTGAAGGTAGGCAATGCTCGTTTAGC | |
| HA/FLAG-p53 | **F**: cgcGGATCCatggaggagccacagtcagac | **R**: acgcGTCGACtcagtccgagtcaggcccctc |
| p53-BHK21-sgRNA-1 | **F**: caccgCATCGAGCTCCCTCTGAGCC | **R**: aaacGGCTCAGAGGGAGCTCGATGc |
| p53-BHK21-sgRNA-2 | **F**: caccgTGTCAGAGAATGTTACAGGC | **R**: aaacGCCTGTAACATTCTCTGACAc |
| p53-PK15-sgRNA-1 | **F**: caccgGCAGATCGTTCACTGCTGCC | **R**: aaacGGCAGCAGTGAACGATCTGCc |
| p53-PK15-sgRNA -2 | **F**: caccgCCTTCTCAGAAGACCTACCC | **R**: aaacGGGTAGGTCTTCTGAGAAGGc |
| SEQ-BHK21-p53 | **F**: AGTTAGGTGTCTGTGATCCCT | **R**: TATTTTCAAAAGCCAAGAAAG |
| SEQ-PK15-p53 | **F**: TGGGAAGCACAGACCTATACT | **R**: TGATGGGAAGGATGAGAGGC |
| qPCR-VP1 | **F**: GACAACACCACCAACCCA | **R**: CCTTCTGAGCCAGCACTT |
| qPCR-3D | **F**: ACTGGGTTTTACAAACCTGTGA | **R**: GCGAGCCCTGCCACGGA |
| qPCR-BHK21-ACT | **F**: GAGAAGCTGTGCTATGTTGCCC | **R**: CCACAGGATTCCATACCCAGGA |
| qPCR-PK15-ACT | **F:** TCATGGACTCTGGGGATGGG | **R**: CGCTCCGTCAGGATCTTCAT |
| qPCR-PK15-IFNB-1 | **F**: GGCTGGAATGAAACCGTCAT | **R**: TCCAGGATTGTCTCCAGGTCA |
| qPCR-PK15-MDA-5 | **F**: CAAATGGCGAGATAATCTGCAA | **R**: TTTGAAAGCCACTACATAATTCTTTATTTT |
| qPCR-PK15-RIG-I | **F**: ACTCACTGCCCCAGGTCATT | **R**: GCTTCAGCCTTGTTTTTTGCA |
| qPCR-PK15-TLR3 | **F**: AAAATCTCCAAGAGCTTCTATTAGCAA | **R**: TTGTATTTGATTTGATGACAACTCTAATCTTT |
| qPCR-Mice-IFNB-1 | **F**: AGTTACACTGCCTTTGCC | **R**: GTTGAGGACATCTCCCAC |
| qPCR-Mice-CCL4 | **F**: CAGCCCTGATGCTTCTCACT | **R**: GGGAGACACGCGTCCTATAAC |
| qPCR-Mice-CCL5 | **F**: GAAGGAACCGCCAAGTGTGT | **R**: CAGGACCGAGTGGGAGTAGG |
| qPCR-Mice-TNF | **F**: CCCACGTCGTAGCAAACCAC | **R**: CTTTGAGATCCATGCCGTTGG |
| qPCR-Mice-CXCL2 | **F**: GTCCCTCAACGGAAGAACCA | **R**: CTCAGACAGCGAGGCACATC |
| qPCR-Mice-IL6 | **F**: GTCCTTCCTACCCCAATTTCCA | **R**: TAACGCACTAGGTTTGCCGA |
| qPCR-Mice-TNFAIP3 | **F**: GAAAGACGTGCACACCCCC | **R**: GTTCAGCCATGGTCCTCGAC |
| qPCR-Mice-IRF3 | **F**: TCCAACAGCCAGCCTATCTC | **R**: AGAATAACCACCAGCCTAGACG |
| Tp53-Mice-P1 | AGTTCTGCCACGTGGTTGGT |  |
| Tp53-Mice-P2 | GTCTCCTGGCTCAGAGGGAG |  |
| Tp53-Mice-P3 | CAGAGGCCACTTGTGTAGCG |  |
